# Global Trends in Cadaver Donation and Medical Education Research: Bibliometric Analysis Based on VOSviewer and CiteSpace

**DOI:** 10.2196/71935

**Published:** 2025-08-18

**Authors:** Xianxian Zhou, Hua Xiong, Yi Wen, Fang Li, Dexi Hu

**Affiliations:** 1 Yiyang Central Hospital, No. 118, Kangfu North Road, Heshan District, Yiyang City, 413000, China, 86 13707378768

**Keywords:** cadaver donation, medical education, bibliometric analysis, citespace, VOSviewer, medical knowledge, medical training, medical student

## Abstract

**Background:**

The cadaver serves as a crucial resource in medical education, research, and clinical practice, as well as a vital foundation for fundamental medical experimental teaching.

**Objective:**

This study aims to use bibliometric analysis to create a knowledge map of cadaver donation in medical education, identify global trends, anticipate future research directions, and offer a foundation for upcoming investigations.

**Methods:**

Articles and review papers concerning cadaver donation and medical education, with a final search cutoff of January 10, 2025, were systematically retrieved from the Web of Science Core Collection database. Two reviewers carefully examined the initial set of articles based on titles and abstracts to exclude irrelevant ones. A quadratic regression model was used to examine the annual publication data. The model’s goodness of fit was assessed using the *R*^2^ value, and the statistical significance of the findings was determined through the *P* valu. The selected publications were then analyzed and visualized for country, institution, author, reference, journal, and keywords using CiteSpace 6.3R3, VOSviewer 1.6.19, and the Online Analysis Platform of the Literature Metrology Database.

**Results:**

The quadratic regression model yielded the equation Y=0.1586X²−633.9X+633395, indicating a substantial increase in the number of publications over time (*R*^2^=0.9575, *P*<.05). The model forecasts that the publication count will reach 107 by 202. This upward trend is statistically significant, highlighting a notable rise in research interest and activity within this field over time. The United States was a major contributor, accounting for 21.2% (303/1114) of all publications. In terms of continents and faiths, Europe and Christianity contributed the most, while McGill University and The University of Sydney were the leading institutions. Prominent authors in this field included De Caro Raffaele, Macchi Veronica, Porzionato Andrea, Stecco Carla, and Dhanani Sonny. The most frequently cocited reference was “Bodies for Anatomy Education in Medical Schools: An Overview of the Sources of Cadavers Worldwide.” The journal Anatomical Sciences Education published the most articles in this area and received the highest citation count. Cluster analysis of keywords revealed that “kidney transplantation,” “gross anatomy education,” and “brain death” were key research topics, while burst analysis of keywords identified “public perception” and “anatomical science” as emerging areas of investigation.

**Conclusions:**

This research presents a distinctive bibliometric approach to cadaver donation within medical education, setting it apart from previous studies by delivering an extensive global overview of trends and influential contributors in this domain. The results emphasize the increasing global interest and collaborative efforts surrounding cadaver donation, while also offering fresh perspectives on emerging topics like public perception and anatomical sciences. This paper serves as an important reference for researchers, policymakers, and educators, supporting the development of future strategies to enhance cadaver donation programs and further medical education.

## Introduction

Cadaver donation plays a vital role in enhancing medical education and training by offering students valuable opportunities for hands-on learning in anatomy, surgery, and other medical fields [1,2]. As global health care systems encounter growing challenges such as organ shortages, ethical issues, and the continuous evolution of medical practices, the demand for cadaver donations has significantly increased [3,4]. Incorporating cadaveric resources into medical education improves students’ learning outcomes by providing realistic, immersive experiences that boost their practical skills and understanding of human anatomy [5].

However, it is essential to recognize the differences in students’ learning styles, as these variations influence their capacity to effectively process and retain information. Some students may benefit more from direct, hands-on cadaveric dissection, while others may find virtual reality (VR) or other digital tools more effective for enhancing their grasp of anatomical structures. In light of the need to accommodate diverse learning styles—especially during periods of remote learning or pandemic conditions—adopting a variety of teaching methods becomes increasingly essential. Research highlights that individual learning preferences have a significant impact on study duration and academic success, reinforcing the need for flexible and adaptive teaching strategies in medical education [6].

Although cadaver-based learning plays a crucial role, research on cadaver donation in medical education is still scattered, lacking a thorough examination of global trends in this area. Existing literature primarily focuses on regional views, ethical dilemmas, and the educational outcomes associated with cadaver-based teaching methods [7-9]. Yet, there is a lack of a broader, global perspective on the patterns, growth, and impact of cadaver donations in medical education. This research aims to fill this gap by conducting a bibliometric analysis of publications related to cadaver donation and medical education, using the advanced analytical tools VOSviewer and CiteSpace. Specifically, the study seeks to address the following research question: What are the global trends, institutional contributions, and emerging themes in the research on cadaver donation and medical education?

The significance of this study lies in its potential to inform future research and policy decisions in medical education. By systematically mapping the academic landscape, identifying key authors, institutions, countries, and themes, this research will offer valuable insights into the current state of the field and pinpoint areas in need of further investigation. Furthermore, understanding global trends in cadaver donation can assist educational institutions and policymakers in better aligning their strategies with best practices, ensuring the ongoing relevance and effectiveness of cadaver-based learning in the ever-changing landscape of medical education.

## Methods

### Data Collections

**Figure 1.**
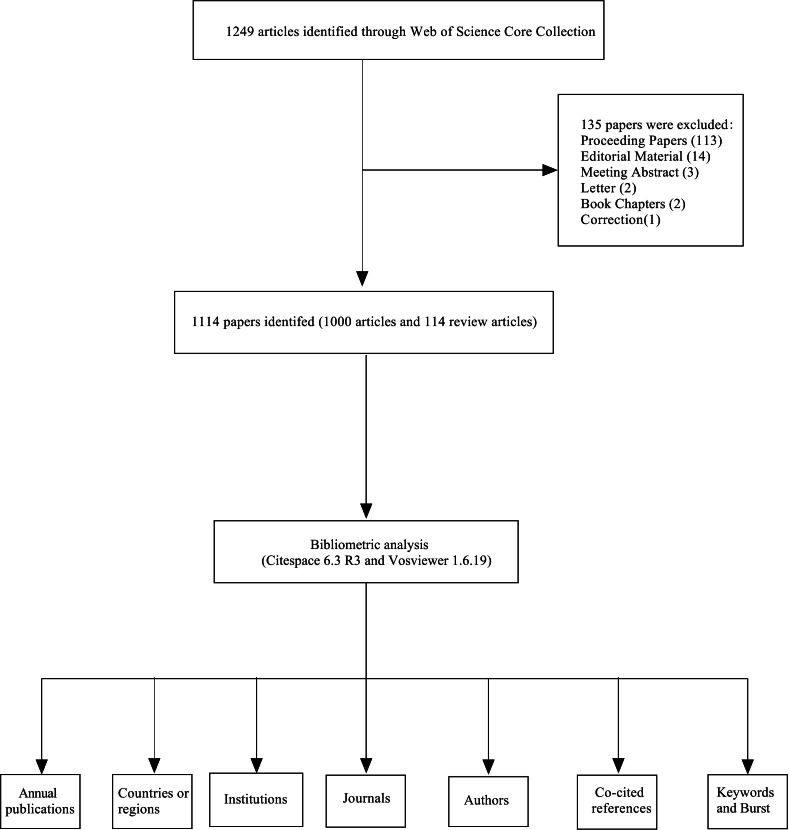
Flowchart for the analysis of cadaver donation and medical education.

Data were collected on January 10, 2025, from the Web of Science Core Collection (WoSCC) database, which includes the Science Citation Index Expanded, Social Sciences Citation Index, Arts and Humanities Citation Index, Emerging Sources Citation Index, Current Chemical Reactions, and Index Chemicus. The search focused on publications related to the keywords “cadaver donation” and “medical education.” The language was limited to English, and the article types were restricted to research articles and reviews. Each record contained details such as the title, author, keywords, abstract, year, institution, citations, and other pertinent information. A detailed explanation of the search methodology, including both inclusion and exclusion criteria, is provided in Figure 1.

### Bibliometrics and Visualization Analysis

The search results were subsequently examined using CiteSpace (Version 6.3R3, Drexel University, Chaomei Chen), VOSviewer (Version 1.6.19; Leiden University), and the Online Analysis Platform of Literature Metrology. CiteSpace, a visual analysis tool developed by Chaomei Chen, was used to analyze the total number of relevant papers, annual trends, keyword frequency, and centrality. This software provided a more accessible and intuitive way to examine the structure, patterns, and distribution of knowledge within the subject. A scientific knowledge map was used to identify research hotspots, advancements, and the current state of the field [10]. VOSviewer, a tool designed for document data analysis, facilitated the study of countries, institutions, authors, journals, keywords, and the co-occurrence knowledge graph for countries, institutions, journals, and publications. In the knowledge graph, each node represented an individual element, with the connection width between nodes reflecting collaboration strength, the node size indicating the number of publications, and larger nodes representing more frequent contributions [11]. The Online Analysis Platform of Literature Metrology was used to analyze the number of publications per country over different years and their collaborative efforts.

### Quadratic Regression Model

A quadratic regression model was applied to fit the publication data. The model is represented by the equation:


(1) $$Y_{t}=\beta_{0}+\beta_{1}t+\beta_{2}t^{2}+\varepsilon_{t}$$

where:

*Y*_t_ is the number of publications in year t.

*t* is the time variable (year).

*β*_0_, *β*_1_, and *β*_2_are the regression coefficients.

*ε_t_* is the error term.

This model allows for both linear and curved trends, which is useful for identifying whether the number of publications has accelerated or slowed over time.

#### Goodness of Fit

The goodness of fit was assessed using the *R*^²^ value. *R*^²^ shows how well the model explains the variation in the publication data. A higher *R*^²^ indicates a better fit, meaning the model accurately reflects the trends in the data.

#### Statistical Significance

To determine the statistical significance of the results, we looked at the *P* value for each regression coefficient. The *P* value tests whether each variable (eg, time or its squared term) significantly affects the number of publications.

A *P* value less than .05 indicates that the coefficient is statistically significant, meaning the variable has a real impact on publication trends.

A *P* value greater than .05 suggests that the variable is not significantly influencing the trend.

The articles for this study were obtained in .txt format from the Web of Science database. Two expert researchers reviewed the title, keywords, and abstract of each paper, applying inclusion and exclusion criteria to screen the documents. In cases of disagreement or uncertainty about a paper’s inclusion, a third reviewer made the final decision through discussion. Initially, 1249 papers were identified, and after excluding 135 papers that were irrelevant to the study’s focus, 1114 papers were retained.

### Ethical Considerations

This study did not involve the collection of new data from humans or animals. All the data used in the bibliometric analysis were sourced from the Web of Science Core Collection, and therefore, ethical approval and participant consent are not required.

## Results

### Annual Publications

**Figure 2.**
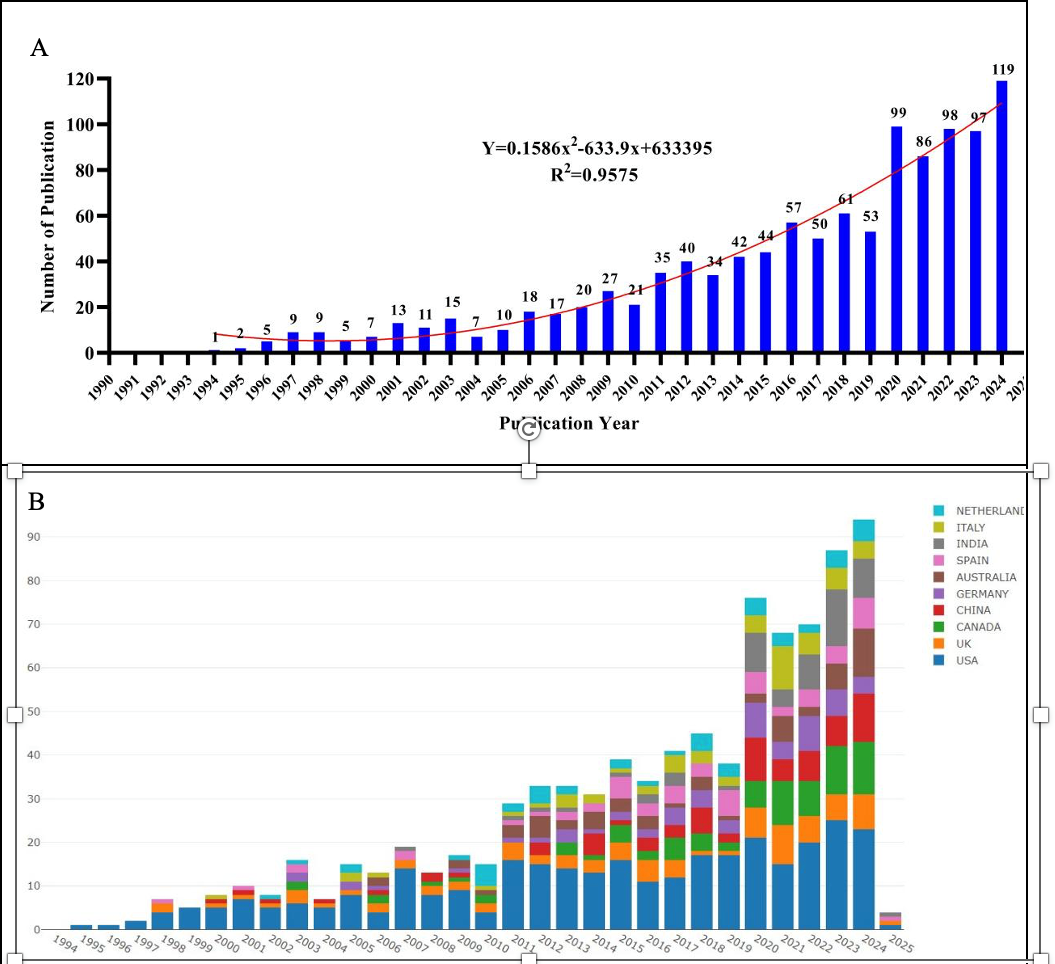
(A) Number of annual publications on cadaver donation and medical education; (B) Number of annual publications and growth trends of the top 10 countries/regions.

Among the 1114 papers on cadaver donation and medical education, the earliest publication dates back to 1994 [12]. Since then, the number of publications has steadily increased each year (Figure 2A). The growth trend of annual publications aligns with the fitted curve: y=0.1586x² – 633.9x + 633395 (*R*^²^=0.9575, *P*<.05). According to this model, it is projected that around 107 papers will be published in this field in 2025.

As part of our thorough analysis, we aimed to identify the countries and regions that have significantly influenced the development of research within this interdisciplinary field. Our geographic analysis, shown in Figure 2B, features a bar chart highlighting the top 10 countries and regions based on their total number of published articles. The United States stands out as a leading contributor to this field, with a considerable and consistently increasing volume of publications.

### National Analysis

**Figure 3.**
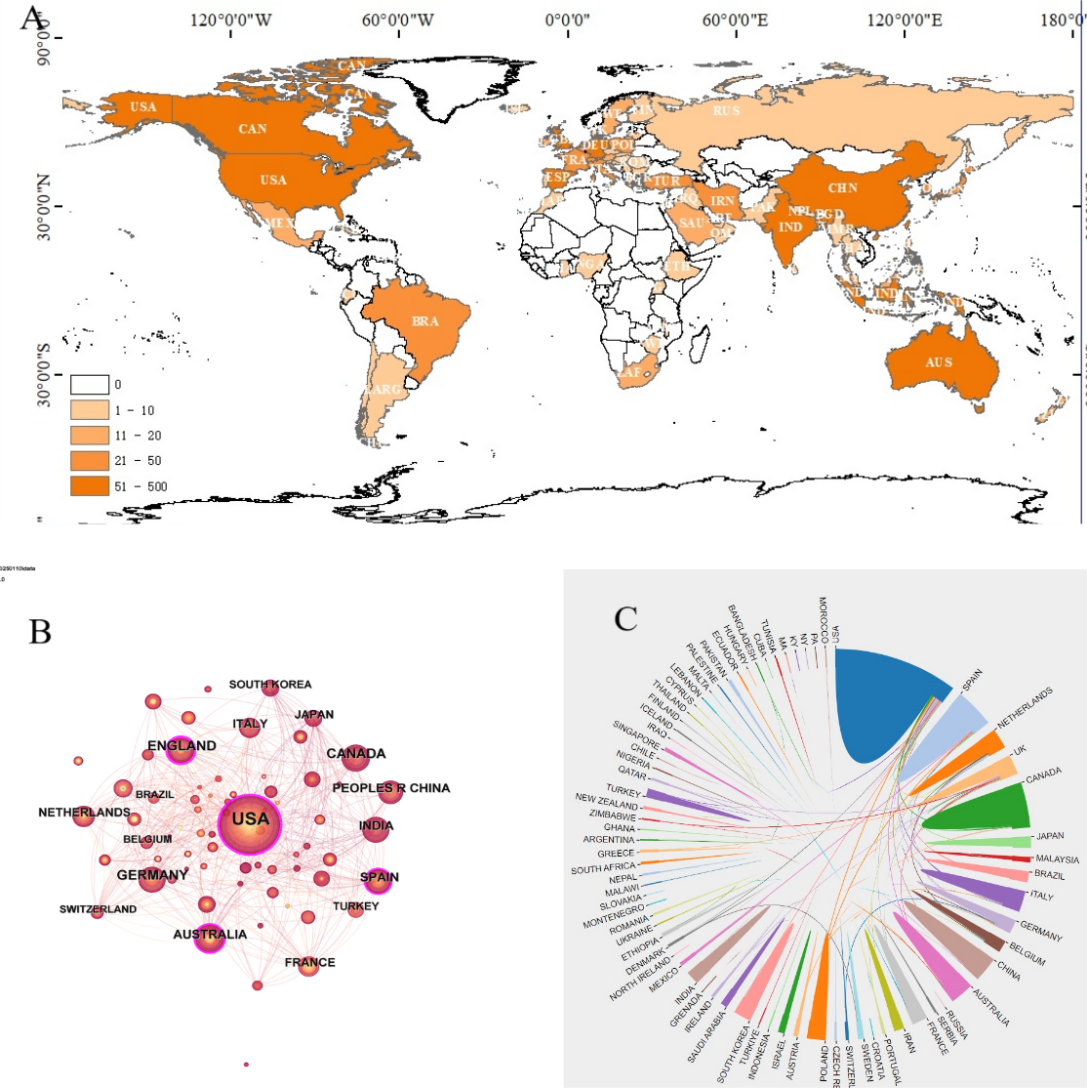
(A) Distribution of countries engaged in cadaver donation and medical education research; (B) The network of co-country co-occurrence; (C) The network map of cooperation between countries or regions.

A total of 1114 publications on cadaver donation and medical education were contributed by 81 countries or regions (Figure 3A). The top 10 countries or regions with the highest number of publications are listed in Table 1. Among these, the United States produced the most original articles, followed by the United Kingdom and Canada. Together, the top 3 accounted for over a third of the total publications. Notably, India and China are the only developing countries in the top 10. The research network map among countries or regions revealed a high density (n=81, E=470, density=0.1451; Figure 3B), indicating strong cooperation between nations. Figure 3C highlights frequent collaborations between the United States and Poland, Spain and Poland, and Germany and Switzerland.

**Table 1.** The top 10 countries and institutions of cadaver donation and medical education.

Country	Documents	Citations	Organization	Documents	Citations
United States	303	9352	McGill University	20	754
United Kingdom	79	2488	University of Sydney	18	735
Canada	74	1658	University of Pittsburgh	16	633
Germany	74	2080	University of Otago	15	444
Australia	57	1388	University of Ottawa	15	286
India	53	1083	Harvard University	14	786
Peoples Republic of China	53	761	University of Montreal	14	74
Spain	53	1458	New York University	13	252
Netherlands	46	1326	Johns Hopkins University	11	674
Italy	46	1050	Canadian Blood Serv	11	172

### Continent Analysis

In this study, we classified medical education institutions involved in body donation research by continent: Asia, Europe, Oceania, North America, South America, and Africa. South America had no participating institutions in this study. The analysis revealed that European medical education institutions had the highest involvement, contributing 774 institutions. North America followed with 501 institutions, while Asia contributed 413 institutions. Oceania had 75 institutions, and South America and Africa had significantly fewer, with 57 and 32 institutions, respectively. These findings highlight the uneven distribution of research across different continents, with Europe and North America leading in terms of medical education institutions (Multimedia Appendix 1).

### Religion Analysis

To examine the influence of religious affiliation on medical education institutions engaged in cadaver donation, we categorized the institutions according to their religious ties. The analysis revealed that medical schools with Christian affiliations were the most numerous, comprising 1441 institutions. Islamic-affiliated institutions ranked second, with 130 institutions reporting such affiliations. Confucian-affiliated institutions followed in third place with 102, while Buddhist institutions accounted for 89. Hindu-affiliated institutions numbered 71, and Jewish-affiliated institutions were the least represented, with only 19 institutions (Multimedia Appendix 2).

### Institutional Analysis

**Figure 4.**
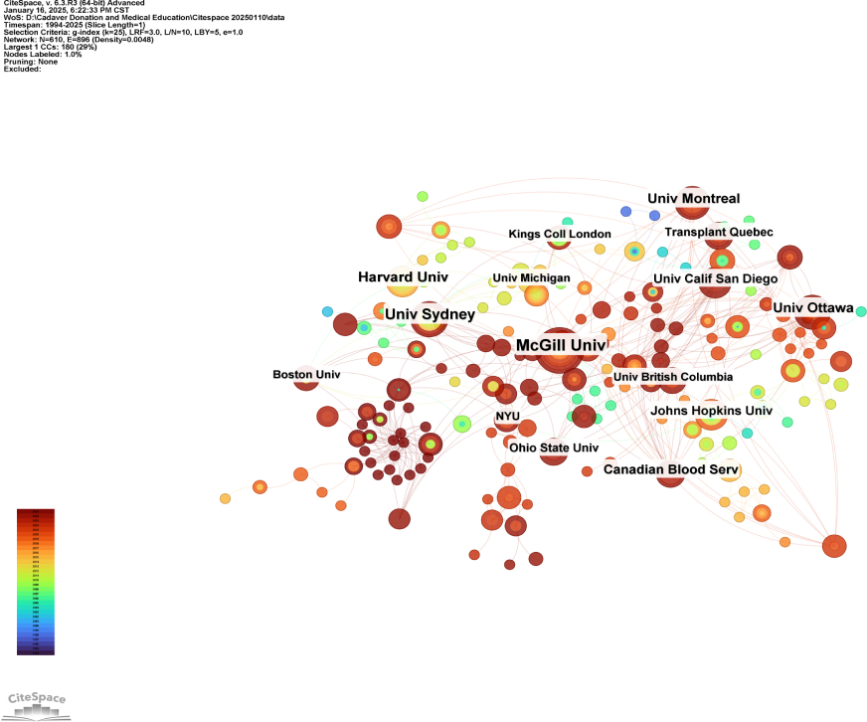
The network of coinstitutions co-occurrence.

An institutional analysis from 1994 to 2025 shows that 1852 institutions participated in research on cadaver donation and medical education. McGill University and the University of Sydney led the list with the highest number of publications, each contributing at least 18 papers. They were followed by the University of Pittsburgh, University of Otago, and University of Ottawa, which contributed 16, 15, and 15 papers, respectively (Table 1). The institutions with the highest citation counts were Harvard University (786 citations), McGill University (754 citations), and the University of Sydney (735 citations). In terms of centrality, McGill University and the University of Sydney ranked first and second, respectively (Figure 4).

### Author Analysis

**Figure 5.**
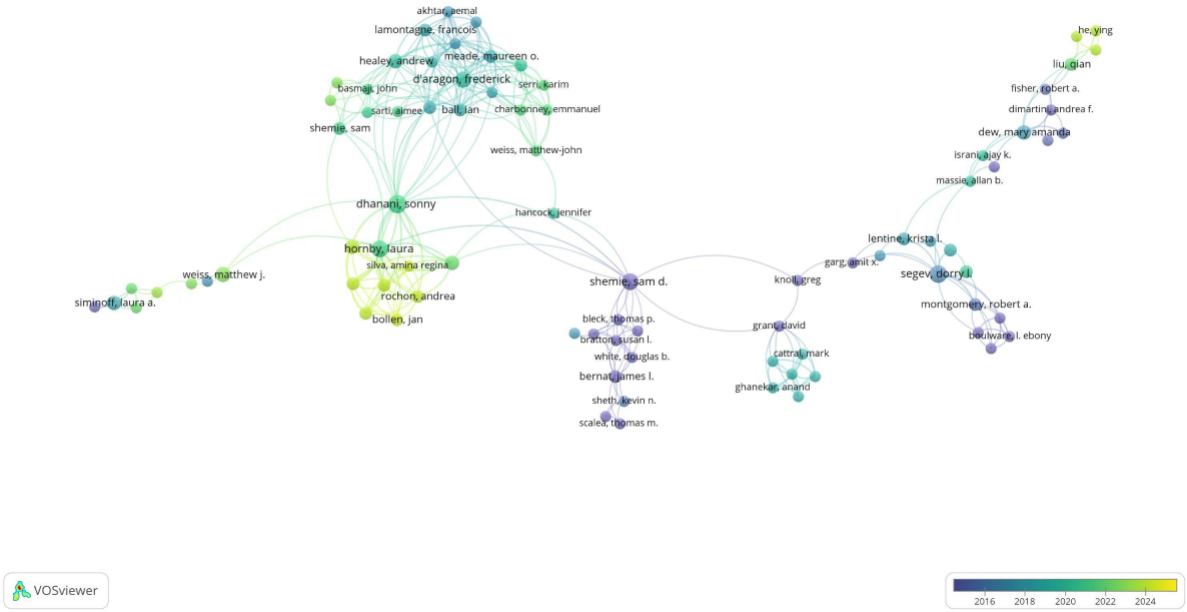
The overlay map of coauthors in cadaver donation and medical education.

Among this group, a subset of 520 authors emerged as particularly productive, with each having published 5 or more articles (T ≥2). This subset was used to create an author network map, as shown in Figure 5. In addition, an overlay visualization map was developed by considering the 84 coauthors of these 520 authors. This knowledge map serves as a visual representation that clearly illustrates high-frequency coauthor collaborations across various years.

In this map, the size of each node reflects the frequency of co-occurrence among authors, while the connecting lines represent coauthor relationships. The total strength of coauthorship connections for each of the 84 authors was measured. The authors with the highest number of publications (n=9) were De Caro, Raffaele; Macchi, Veronica; Porzionato, Andrea; Stecco, Carla; and Dhanani, Sonny (Table 2). Cocited authors are those who frequently appear together in multiple publications. In this field, 20,889 co-cited authors were identified, with 134 of them having more than 20 citations (T ≥ 20). The most frequently cocited author was Jones DG (n=232), followed by Siminoff LA (n=177), Cornwall J (n=159), Bolt S (n=133), and Boulware Le (n=126). Other leading authors had cocitation counts ranging from 89 to 125, as shown in Table 2.

**Table 2.** The top 10 coauthors and co-cited authors of cadaver donation and medical education.

Rank	Coauthor	Documents	Citations	Co-cited author	Citations
1	De Caro, Raffaele	9	219	Jones DG	232
2	Macchi, Veronica	9	219	Siminoff LA	177
3	Porzionato, Andrea	9	219	Cornwall J	159
4	Stecco, Carla	9	219	Bolt S	133
5	Dhanani, Sonny	9	78	Boulware Le	126
6	Segev, Dorry L	8	319	Ghosh SK	125
7	Hornby, Laura	7	171	Riederer BM	118
8	Shemie, Sam D	7	640	Winkelmann A	115
9	Balta, Joy Y	6	24	Ríos A	107
10	D'aragon, Frederick	6	36	Hildebrandt S	89

### References Analysis

**Figure 6.**
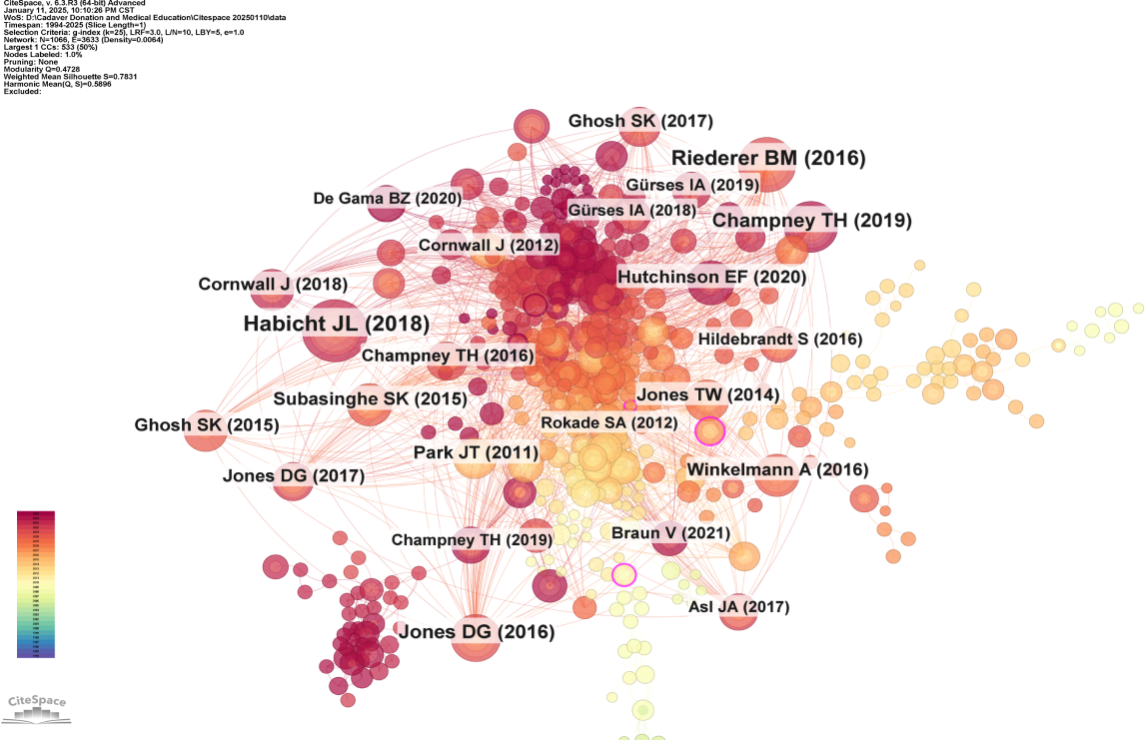
The network of co-cited references in cadaver donation and medical education research.

**Figure 7.**
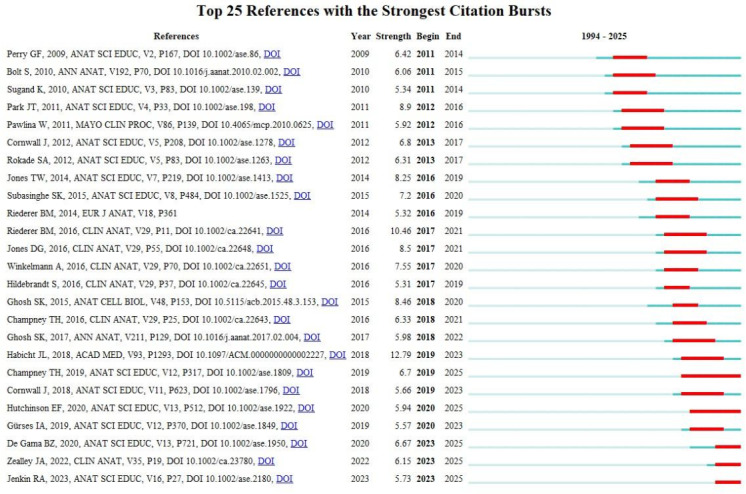
The top 25 references with the strongest citation bursts (sorted by the beginning year of burst) [13-37].

CiteSpace was used to analyze cocited references. Figure 6 and Table 3 display the top 10 co-cited references with the highest frequency and betweenness centrality. The co-citation network in the field of cadaver donation and medical education research consisted of 29,078 references, 1066 nodes, and 3633 links. Figure 7 [13-37] presents the top 25 references with the most significant citation bursts, which indicate emerging trends or growing interest in the subject. In general, references with higher co-citation rates are associated with stronger citation bursts. The citation burst for the article “Age Modulates Attitudes to Whole Body Donation Among Medical Students” authored by Perry GF et al [13]. and published in *Anatomical Sciences Education*, began in 2011 with a burst strength of 6.42. This study explores how age affects medical students’ views on whole body donation, using Likert-type questionnaires administered to first-year graduate-entry students before and after their dissection experiences.

**Table 3.** The top 10 co-cited references of cadaver donation and medical education.

Rank	First author	Country	Frequency	Centrality	Year	Source
1	Habicht JL [14]	Germany	38	0.01	2018	*Academic Medicine*
2	Riederer BM [15]	Switzerland	32	0.06	2016	*Clinical Anatomy*
3	Champney TH [16]	United States	27	0.01	2019	*Anatomical Sciences Education*
4	Jones DG [17]	New Zealand	23	0.02	2016	*Clinical Anatomy*
5	Hutchinson EF [18	South Africa	19	0.02	2020	*Anatomical Sciences Education*
6	Subasinghe SK [19]	New Zealand	18	0.02	2015	*Anatomical Sciences Education*
7	Park JT [20]	South Korea	18	0.02	2011	*Anatomical Sciences Education*
8	Cornwall J [21]	New Zealand	17	0.01	2018	*Anatomical Sciences Education*
9	Ghosh SK [22]	India	17	0	2015	*Anatomy and Cell Biology*
10	Jones TW [23]	United States	17	0.02	2014	*Anatomical Sciences Education*

### Journals Analysis

We used VOSviewer to perform cocitation and cocited journal analyses, enabling us to identify the most prominent and influential journals in the field. The analysis revealed that 1114 papers were published across 474 academic journals (with a minimum citation count of 20 per source). *Anatomical Sciences Education* had the highest number of citations (n=2586), followed by journals such as *American Journal of Transplantation*, *Transplantation*, *Clinical Anatomy*, *Critical Care Medicine*, *Clinical Transplantation*, *Nephrology Dialysis Transplantation*, *Annals of Anatomy-Anatomischer Anzeiger*, *Social Science & Medicine*, and *Human Reproduction Open*. Among the top 10 journals, five had an impact factor greater than 5 (Table 4).

**Figure 8.**
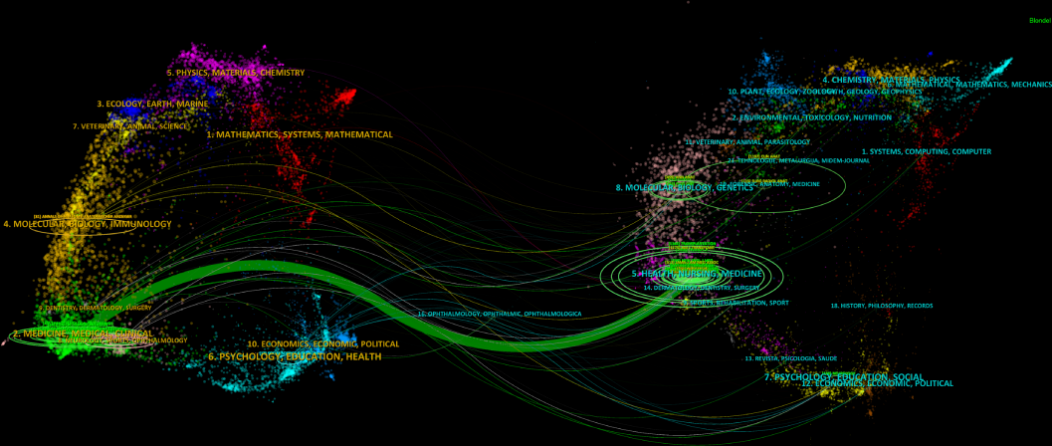
The dual-map overlay of journals related to cadaver donation and medical education research. The citing journals were at left, the cited journals were on the right, and the colored curve represents the reference path.

Among the 10,465 cocited journals, five had over 1000 citations. As presented in Table 5, *Anatomical Sciences Education* led with the highest number of co-citations (n=1946), followed by *Transplantation and Clinical Anatomy*. The dual-map overlay of journals illustrates the distribution of topics within academic journals (Figure 8). A prominent citation pathway, shown in green, was identified, indicating that studies published in Medicine or Medical or Clinical journals were predominantly cited by articles from Health or Nursing or Medicine journals.

**Table 4.** The top 10 journals of cadaver donation and medical education.

Rank	Journal	Citations	Impact factor (2023)	JCR division	Country
1	*Anatomical Sciences Education*	2586	5.2	Q1	United States
2	*American Journal of Transplantation*	999	8.9	Q1	United States
3	*Transplantation*	933	5.3	Q1	United States
4	*Clinical Anatomy*	842	2.3	Q4	United States
5	*Critical Care Medicine*	772	7.7	Q1	United States
6	*Clinical Transplantation*	628	1.9	Q4	United States
7	*Nephrology Dialysis Transplantation*	561	4.8	Q2	England
8	*Annals of Anatomy-Anatomischer Anzeiger*	543	2.0	Q3	Germany
9	*Social Science & Medicine*	517	4.9	Q1	England
10	*Human Reproduction Open*	451	8.3	Q1	England

**Table 5.** The top 10 co-cited journals of cadaver donation and medical education.

Rank	Co-cited journal	Co-citations	Impact factor (2023)	JCR division	Country
1	*Anatomical Sciences Education*	1946	5.2	Q1	United States
2	*Transplantation*	1347	5.3	Q1	United States
3	*Clinical Anatomy*	1198	2.3	Q2	United States
4	*American Journal of Transplantation*	1177	8.9	Q1	United States
5	*Transplantation Proceedings*	1068	0.8	Q4	United States
6	*New England Journal of Medicine*	590	96.2	Q1	United States
7	*Journal of the American Medical Association*	515	63.1	Q1	United States
8	*Annals of Anatomy-Anatomischer Anzeiger*	460	2.0	Q2	Germany
9	*Liver Transplantation*	445	4.7	Q1	United States
10	*Clinical Transplantation*	430	1.9	Q2	Denmark

### Keywords Analysis

**Figure 9.**
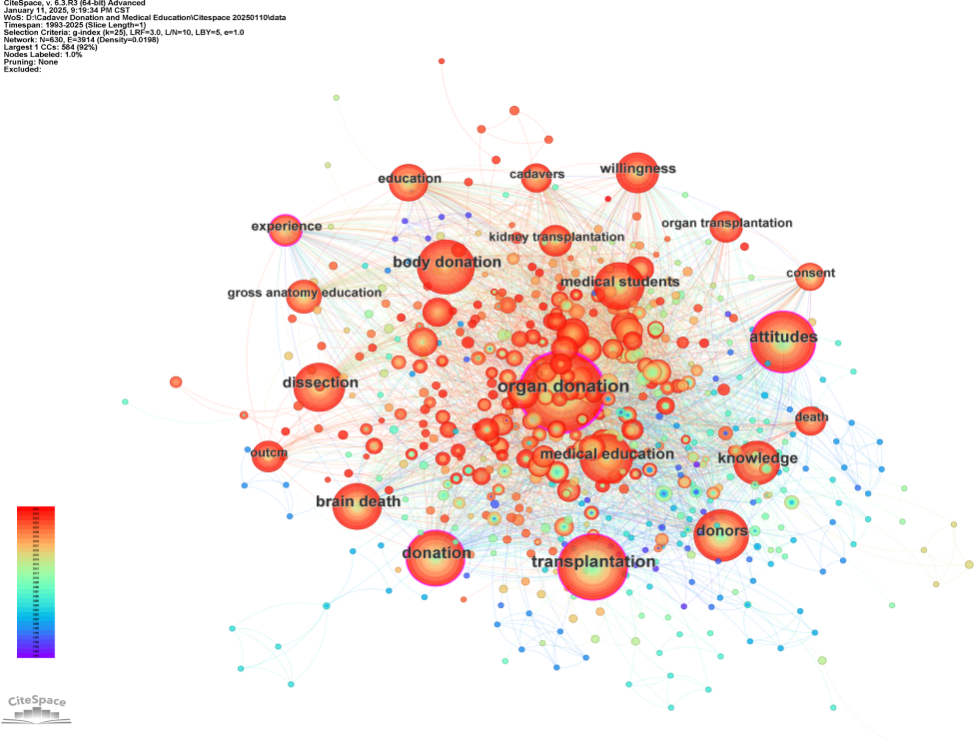
The network of keywords co-occurrence.

Keywords act as concise representations of articles, capturing their essential themes. Keywords that occur frequently and are centrally positioned often highlight current and significant areas of research within a discipline. We examined publications by segmenting them into 1-year intervals and selecting the top 20 most cited or frequent items from each period (Figure 9). The development of relevant research is illustrated through a hybrid network where co-occurring keywords from titles and abstracts shape the representation. Various maps display nodes representing keywords, with node size reflecting their frequency of occurrence or citation, and node color indicating the years of occurrence or citation. Red circles on the map denote keyword bursts, signaling rapid increases in publication frequency. By analyzing keyword frequency and centrality, key research frontiers can be identified.

The keyword network map consists of 630 nodes connected by 3914 links. Significantly, the term “organ donation” stands out due to its high frequency and centrality, indicating its considerable relevance and influence within the research field (Figure 9). Similarly, terms like “Transplantation” and “Attitudes” also appear as high-frequency keywords, highlighting their importance and prominence in the context of the study. Table 6 presents the top 20 keywords with the highest frequency and centrality.

**Table 6.** Top 20 keywords of cadaver donation and medical education research in terms of frequency and centrality.

Rank	Keyword	Freq (count)	Centrality	Rank	Keyword	Freq (count)	Centrality
1	Organ Donation	287	0.26	11	Medical Students	89	0.04
2	Transplantation	181	0.16	12	Willingness	71	0.06
3	Attitudes	156	0.12	13	Experience	58	0.13
4	Donation	147	0.19	14	Education	54	0.05
5	Body Donation	122	0.02	15	Gross Anatomy Education	47	0.01
6	Brain Death	117	0.09	16	Organ Transplantation	47	0.03
7	Knowledge	104	0.03	17	Death	46	0.07
8	Donors	98	0.1	18	Cadavers	45	0.03
9	Medical Education	94	0.02	19	Outcm	45	0.03
10	Dissection	91	0.03	20	Kidney Transplantation	44	0.09

### Keyword Timeline View

Keyword cluster analysis is an effective method for identifying key research topics within a particular field. In this study, CiteSpace was employed to perform a cluster analysis of keywords related to cadaver donation and medical education. The number of clusters was determined by the size of each cluster, with the largest one assigned the label #0.

**Figure 10.**
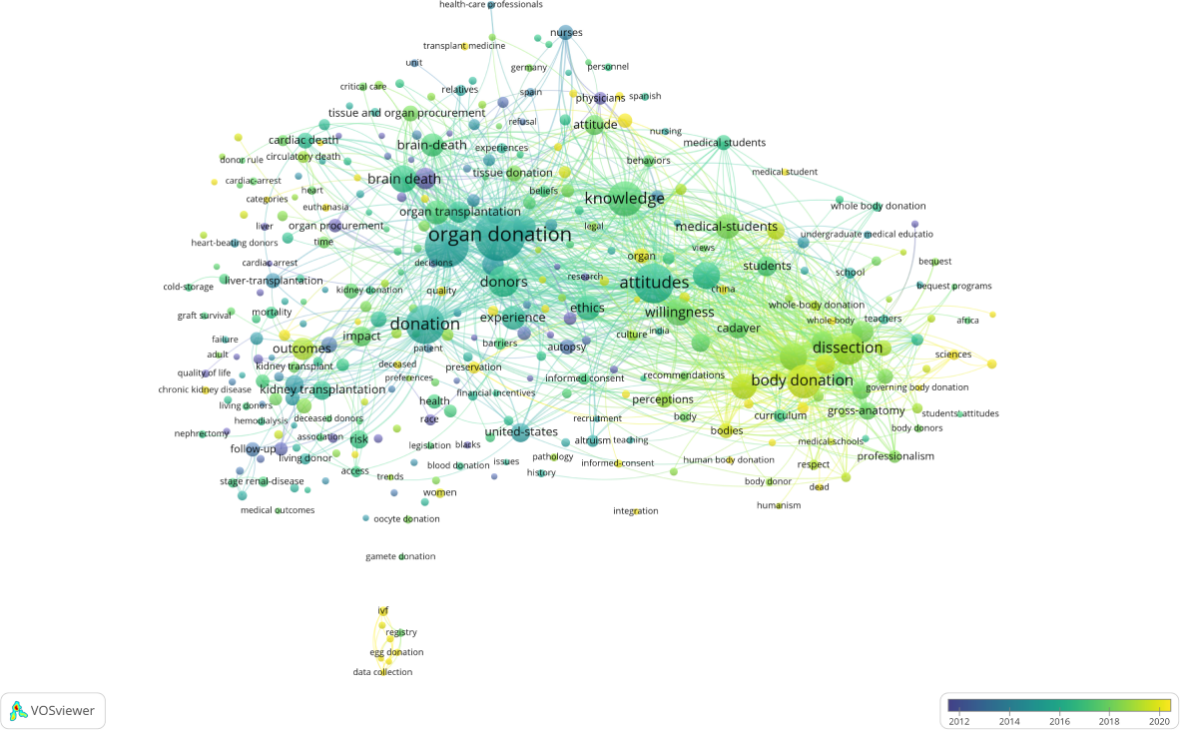
Keywords are clustered on the time scale and output as map.

The analysis produced 6 clusters, which were then examined through a timeline view in CiteSpace. These clusters comprised #0 kidney transplantation, #1 gross anatomy education, #2 brain death, #3 organ donation, #4 body donation, and #5 complications. Figure 10 illustrates the timeline view derived from this cluster analysis.

The research field, when viewed through the lens of emerging trends and development status, is categorized into two areas based on the co-occurrence analysis: (1) Anatomy education (#1, #3, #4) and (2) Clinical application (#0, #2, #5). In addition, the analysis indicated a steady and increasing interest in clusters #0, #1, and #4 in recent years. This highlights the importance of body donation in medical education and research, offering students valuable practical experience in anatomy. It aids in the enhancement of surgical skills, supports progress in organ transplantation, and contributes to medical studies that could lead to new treatments and better health care. Therefore, body donation plays a vital and altruistic role in advancing medical science and improving public health.

In summary, the cluster analysis and timeline visualization offer important perspectives on research trends, thematic development, and the growing significance of specific keywords in the fields of cadaver donation and medical education.

### Research Frontier

The burst keyword analysis conducted with CiteSpace is a useful method for identifying rapidly emerging terms, referred to as burst keywords. These keywords reflect significant attention within the academic community and serve as key indicators of evolving research trends. Through this analysis, 25 representative keywords were chosen from a total of 3947 keywords to pinpoint the main research hotspots. The keywords were organized based on their duration, start time, and burst intensity. (The green line represents the period from 1993 to 2025, while the red line indicates the duration of each burst keyword.)

The term “public attitudes” emerged as the first burst keyword in 1994 (Figure 13A), suggesting that the public’s perception of cadaver donation influences the willingness to donate, thereby affecting the development of medical education and research from the outset. “Body donation” exhibited the highest burst intensity (Figure 13B), highlighting its essential role in cadaver donation, as it provides vital specimens for practical anatomical studies and research in medical education. In addition, “public attitudes” demonstrated the longest burst duration (Figure 13C), indicating that the public’s views on cadaver donation, shaped by enduring cultural perceptions, continue to influence its importance in medical education and research.

The analysis revealed 25 burst keywords, which can be grouped into two main categories: (1) Public perception and (2) Anatomical science. These burst keywords showed a higher concentration between 1994 and 2025. When combined with the triangular pattern depicted in the keyword timeline map (generated using VOSviewer to organize keywords over time), it indicates that research on cadaver donation and medical education is increasingly shifting toward more specialized areas (Figure 12).

Recent significant burst keywords include “awareness” (4.68), “organ” (4.46), and “willingness” (4.1), with the numbers in parentheses indicating the burst strength of each term. These keywords emphasize the importance of public awareness and the willingness to participate in cadaver donation, which are crucial in promoting and shaping medical education. They play a key role in fostering critical thinking, offering students ethical hands-on experiences, and enhancing their understanding of anatomy and skill development.

This analysis suggests that the field of cadaver donation and medical education has experienced substantial growth and improvement over the past 2 decades, with increasingly focused and specialized research areas emerging. The earlier concentration of burst keywords may signify a phase of rapid progress and discovery, while the more recent burst keywords highlight the growing emphasis on raising public awareness of cadaver donation to improve medical education. The advancement of medical education depends significantly on public support and understanding, as body donors play a crucial role. The shortage of donors, in particular, presents a major challenge to both transplant medicine and foundational medical training, emphasizing the need for greater acknowledgment of their indispensable contribution. Overall, the burst keyword analysis offers valuable insights into the evolution and current state of research in cadaver donation and medical education.

**Figure 11.**
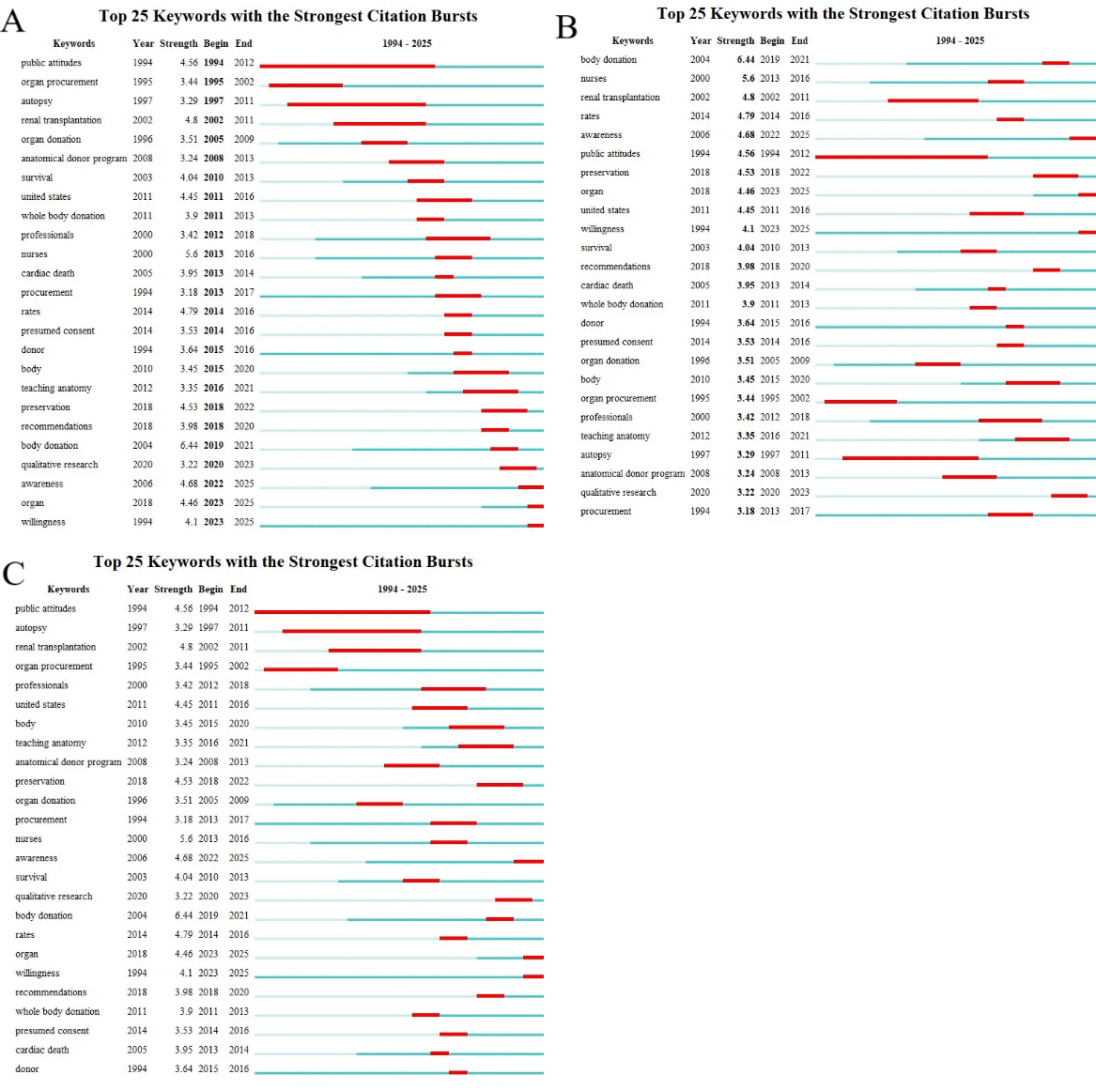
Burst keywords involved cadaver donation research in relation to medical education. (A) Ranking by beginning. (B) Ranking by strengths. (C) Ranking by durations.

**Figure 12.**
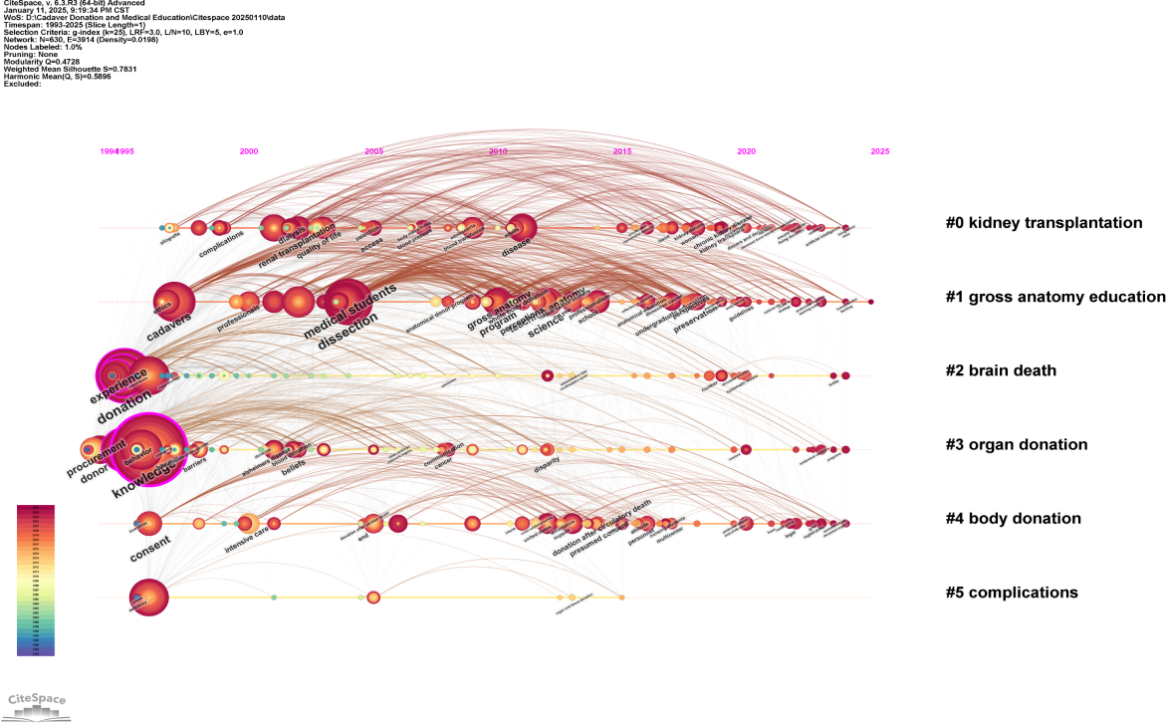
The keywords of cadaver donation and medical education were shown in the keywords timeline view.

## Discussion

### Principal Findings

This research offers an in-depth bibliometric analysis of cadaver donation in medical education, providing fresh perspectives on global trends and contributions within the field. The results indicate a significant rise in research activity over time, with projections suggesting a continued upward trajectory in the coming years. This increasing interest highlights the growing recognition of cadaver donation as an essential resource for medical education, research, and clinical practice. By pinpointing key contributors, influential institutions, and emerging research areas, this study lays a crucial groundwork for future inquiries and emphasizes the collaborative efforts shaping the cadaver donation landscape.

The contribution of this study to the existing body of literature is especially valuable due to its extensive global scope, which surpasses the focus of previous studies in both breadth and depth. Unlike earlier works that typically concentrate on regional or specific aspects [38,39], this analysis offers a more comprehensive and holistic understanding of global cadaver donation trends. In addition, it introduces new perspectives, including emerging topics such as public perception and anatomical sciences, which are increasingly becoming central to discussions on cadaver donation.

In the subsequent sections, we will explore the broader context of cadaver donation in medical education, examine the key research areas advancing the field, and look into potential future developments that could transform the role of cadaver donation in medical practice. This structured approach aims to provide a thorough understanding of the evolving trends and future directions in this vital aspect of medical education.

### General Information

The WOS database is a widely recognized platform that compiles a vast number of scientific research studies [40]. Based on the literature inclusion and exclusion criteria outlined earlier, 1114 articles were ultimately selected for this study. Since 1994, publications related to cadaver donation and medical education have been consistently released, showing a growing trend. This suggests that the field is gaining increasing attention and has the potential to emerge as a prominent area of research in the future. The United States, the United Kingdom, and Canada lead in the number of publications on this topic, with strong collaborative efforts across geographic borders.

This study indicates that Europe and North America lead in research on cadaver donation and medical education, primarily influenced by Christian cultural norms that encourage body donation for medical purposes. In Europe, the origin of Western medicine, cadaver donation became an early component of medical education, fostering significant advancements in these regions. In contrast, other areas, including parts of Asia and the Middle East, have made slower progress in this field due to economic conditions, cultural factors, and religious beliefs. For instance, in some countries, religious taboos limit the acceptance of cadaver donation, leading to delays in related research. Therefore, cultural and religious influences play a crucial role in shaping the development of cadaver donation research worldwide.

McGill University and The University of Sydney are the institutions with the highest number of publications, reflecting their significant contributions to research in cadaver donation and medical education. A total of 5708 authors have contributed to the literature in this research area. The coauthorship and cocitation analysis highlighted the most influential scholars in the fields of cadaver donation and medical education. De Caro, Raffaele; Macchi, Veronica; Porzionato, Andrea; Stecco, Carla; and Dhanani, Sonny were identified as the most productive authors, underscoring their substantial impact on the literature. Strong collaborative ties among authors were observed, with several prominent coauthor partnerships emerging. These collaborations play a crucial role in advancing knowledge and fostering innovation in cadaver donation and medical education. Furthermore, the identification of cocited authors reveals the key researchers and their significant contributions to the field.

Analyzing the cocited references enabled the identification of the most significant and frequently referenced studies in the field. The leading cocited reference was a paper by Habicht JL et al [14], published in ACAD MED, titled “Bodies for Anatomy Education in Medical Schools: An Overview of the Sources of Cadavers Worldwide.” This study examines global sources of cadavers for anatomical education, evaluating the use of body donations and unclaimed bodies while exploring cultural and institutional factors. Another important source was a viewpoint article by Riederer BM [15], titled “Body Donations Today and Tomorrow: What is Best Practice and Why?” published in Clinical Anatomy. This article highlights the significance of body donations for anatomy teaching and research, reviews the current state of donation programs worldwide, and identifies ethical considerations. The author also looks to the future, advocating for the replacement of unclaimed bodies with formal donation programs to improve both ethical standards and educational outcomes.

The majority of the top 10 journals publishing research on cadaver donation and medical education are prestigious journals from the Q1 and Q2 quartiles, including Anatomical Sciences Education, American Journal of Transplantation, and Transplantation. Furthermore, these journals also ranked highly in terms of co-citations.

### Current Research Hotspots

Through keyword co-occurrence and cluster analysis, this study pinpointed key research areas in cadaver donation and medical education, which were primarily categorized into 2 themes: anatomy education and clinical practice.

### Anatomy Education

Cadaver donation plays an essential role in anatomy education, serving as the cornerstone for hands-on anatomical learning [41]. The value of cadavers in providing real-world insight into human anatomy is irreplaceable, offering students the opportunity to understand complex anatomical structures through direct dissection and observation. This experiential learning approach fosters a deeper understanding of the human body, which is crucial for medical students’ clinical training.

However, a significant challenge persists in the availability of cadaveric specimens. Despite the recognized importance of cadaver-based education, there remains a shortage of donated bodies available for anatomical study, particularly in clinical medical programs [42]. This scarcity hampers the ability to provide sufficient dissection opportunities for students, leading to a gap in practical training. As a result, students often lack the hands-on experience necessary for mastering essential anatomical skills, which can negatively impact their overall learning outcomes.

The limited access to cadavers not only affects anatomy education but also poses a barrier to enhancing the quality of medical education as a whole. The lack of dissection practice diminishes students’ ability to apply theoretical knowledge to clinical scenarios, thereby restricting their development as proficient health care professionals. Furthermore, the global demand for cadavers in medical education continues to grow, intensifying the need for a more robust system to facilitate cadaver donations and ensure equitable access to these critical learning resources [43].

In conclusion, cadaveric donation remains integral to anatomy education, yet the shortage of cadaver specimens represents a significant challenge. Addressing this issue is vital for advancing medical education and enhancing the quality of training for future health care professionals.

### Clinical Practice

The rapid advancement of medical technologies has greatly influenced clinical practice, especially in the field of transplantation. One of the most significant challenges hindering the progress of transplant medicine is the shortage of donor resources [44]. Despite this limitation, significant clinical achievements have been made in corneal and kidney transplantation, demonstrating the profound impact of transplant technologies [45,46]. These advancements have saved thousands of patients suffering from end-stage organ failure, providing them with new hope for survival and improved quality of life.

As medical technology continues to evolve, the focus of research is increasingly shifting toward clinical applications, particularly in the field of transplantation. Cadaver donation plays a pivotal role in this context. Not only does it support the development of anatomy education, but it is also an invaluable resource for advancing clinical practices, particularly in transplantation medicine. Donated bodies provide essential tissue samples for research, leading to improvements in surgical techniques, transplant procedures, and postoperative care.

Furthermore, the use of cadavers in clinical practice indirectly contributes to medical education by providing real-life case studies for students. By integrating cadaver donation into clinical training, medical institutions can equip future health care professionals with the practical experience and knowledge necessary for handling complex transplantation procedures [47]. This, in turn, enhances the quality of clinical education and prepares students to address the challenges of modern medical practice.

In conclusion, cadaver donation is not only crucial for anatomical studies but also plays a vital role in the development of clinical practices, particularly in transplantation. By supporting both medical education and clinical advancements, cadaver donations offer a pathway for saving lives and improving health care outcomes globally.

### Future Frontiers

According to the analysis of keyword emergence, public perception and anatomical science are identified as emerging trends for the future.

### Public Perception

The role of cadaver donation in medical research and education has profoundly influenced the development of modern medicine. For over half a century, cadaver donation programs have served as a cornerstone of anatomical studies and medical training, particularly in the United States [48]. These programs have allowed for significant advancements in medical education, enabling students to learn through direct interaction with human anatomy. However, the extent of cadaver donation for medical purposes remains heavily dependent on public perception, which varies widely across cultures and regions.

In many countries, especially in East Asia, cadaver donation faces significant challenges due to cultural and religious beliefs. Confucian ideals, which emphasize filial piety and the preservation of the body in its natural state, have deeply shaped attitudes toward death and burial practices [49,50]. This cultural framework has made it difficult for many to consider donating their bodies for scientific or educational purposes. As a result, public awareness of and participation in body donation programs remain relatively low in these regions, which, in turn, limits the availability of cadavers for medical education.

Despite these challenges, the importance of cadavers in medical education, particularly in anatomy, cannot be overstated. With the rapid advancements in medical technologies and diagnostic tools, the role of cadaver-based learning remains irreplaceable in developing comprehensive clinical knowledge and skills. As the legal and regulatory frameworks for cadaver donation continue to improve, it is crucial that efforts are made to increase public awareness and understanding of the significance of body donation. Future research should focus on enhancing public education, developing efficient and ethical donation systems, and addressing cultural and religious barriers. By fostering a more informed and supportive public perception, we can ensure that cadaver donation remains a vital resource for the future of medical education and health care.

### Anatomical Science

Anatomy is a foundational subject in medical education, essential for all medical students. It provides students with a direct and comprehensive understanding of the human body’s structure, forming the basis for further studies in physiology, pathology, and surgery [51]. The importance of anatomy extends beyond education; it is integral to clinical practice, diagnosis, and treatment. For example, when interpreting radiological images (eg, x-rays, computed tomographic scans, and magnetic resonance images), physicians rely on their anatomical knowledge to differentiate between normal and abnormal tissue structures [52]. During surgery, surgeons use their understanding of anatomy to navigate organ positions and vascular and neural distributions, ensuring precision and minimizing the risk of damaging vital tissues [53].

In addition to its educational significance, the study of anatomy plays a critical role in shaping a medical student’s ability to grasp fundamental concepts and apply them to real-world scenarios. As the field of medicine advances, anatomical science continues to be indispensable. This is particularly evident in the emerging field of imaging anatomy, where advancements in imaging technologies demand an in-depth understanding of anatomical structures for accurate interpretation and diagnosis. Furthermore, transplant medicine relies heavily on anatomical knowledge, as precise identification of organ structures and their functions is crucial for successful transplantation and postoperative care.

Given these factors, future research in anatomical science should focus on enhancing educational methodologies, particularly in the context of imaging and transplantation. This will ensure that anatomy continues to evolve in alignment with modern medical advancements, ultimately improving patient outcomes and clinical practices.

### Advantages

This study presents several notable strengths that enhance its importance in the field of cadaver donation and medical education. Firstly, the application of bibliometric analysis provides a thorough, data-driven examination of the global research landscape, pinpointing significant trends, key contributors, and emerging areas of focus within the field. By using tools like CiteSpace and VOSviewer, the study delivers detailed visualizations and insights into the evolution of cadaver donation research, making the findings more accessible to future researchers and decision-makers. Second, the comprehensive dataset, which includes articles up to January 2025, ensures that the results reflect the latest and most pertinent advancements in this field. The quadratic regression model’s strong goodness of fit (*R*^2^=0.9575) and statistically significant outcomes (*P*<.05) further validate the robustness of the analysis, offering a dependable forecast of future research trajectories. Finally, the international scope of the study, highlighting contributions from various regions and institutions, provides a well-rounded view of the global cadaver donation research landscape. The identification of emerging topics such as public perception and anatomical sciences demonstrates the study’s potential to influence future research directions and inform the development of cadaver donation and medical education programs worldwide.

### Limitations

Although this study is the first to apply bibliometric methods to conduct a thorough analysis of the research trends and key topics in cadaver donation and medical education, there are some limitations. First, authors often cite recently published journal articles to increase the likelihood of acceptance, which may influence the variety and representativeness of the literature [54]. Second, researchers tend to favor citing highly cited works, potentially overlooking the actual quality and substance of the research, leading to citation bias [55]. Furthermore, this analysis relies solely on the Web of Science core database, which may exclude other significant data sources and introduce the risk of missing valuable information. Finally, only English-language literature was considered, leaving out potentially important findings in other languages. It is also worth noting that high-quality, recent research may not receive adequate citations due to shorter publication cycles, which could result in underrepresentation of its research value.

### Conclusion

In summary, this analysis emphasizes the rapid expansion of cadaver donation research, particularly in Europe and North America, where regions with predominantly Christian populations contribute the most medical education institutions. Notable contributors include the United States, McGill University, and The University of Sydney, which play a pivotal role in shaping the field, reflecting their advanced medical infrastructure and academic commitment. Key research topics such as kidney transplantation, gross anatomy education, and brain death dominate, while emerging areas like public perception and anatomical science present new opportunities for exploration.

This study enriches the literature by offering a comprehensive overview of global trends in cadaver donation research and its influence on medical education, especially in regions with well-established medical traditions. It highlights significant institutions, authors, and themes, serving as a valuable reference for future research. Furthermore, it underscores the importance of international collaboration in advancing the field and advocates for increased public awareness and support for cadaver donation. The findings of this study will inform future policies and research strategies aimed at enhancing medical education and encouraging innovation in teaching methods.

## Supplementary material

10.2196/71935 Multimedia Appendix 1 Distribution of the number of medical education institutions engaged in cadaver donation and medical education across different continents.

10.2196/71935 Multimedia Appendix 2 Distribution of the number of medical education institutions engaged in cadaver donation and medical education across different religions.

## Abbreviations

VR: virtual reality
WoSCC: Web of Science Core Collection
