# Supplementary figures and images for: Global Trends in Cadaver Donation and Medical Education Research: Bibliometric Analysis Based on VOSviewer and CiteSpace
Source: JMIR Med Educ. 2025 Aug 18;11:e71935. doi: 10.2196/71935 (PMC12369992; doi:10.2196/71935)

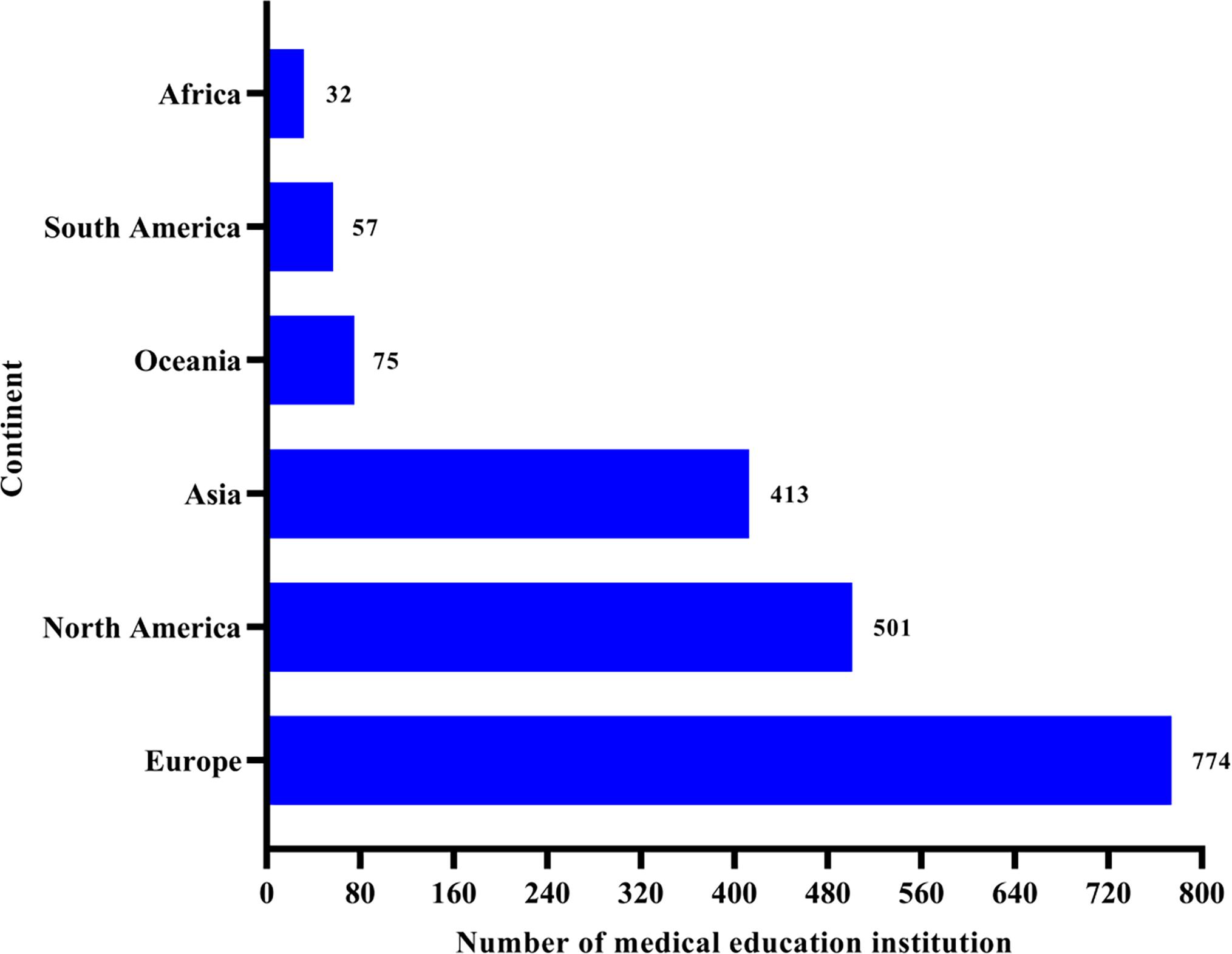

Supplement: Multimedia Appendix 1 [file mededu-v11-e71935-s001.jpeg]

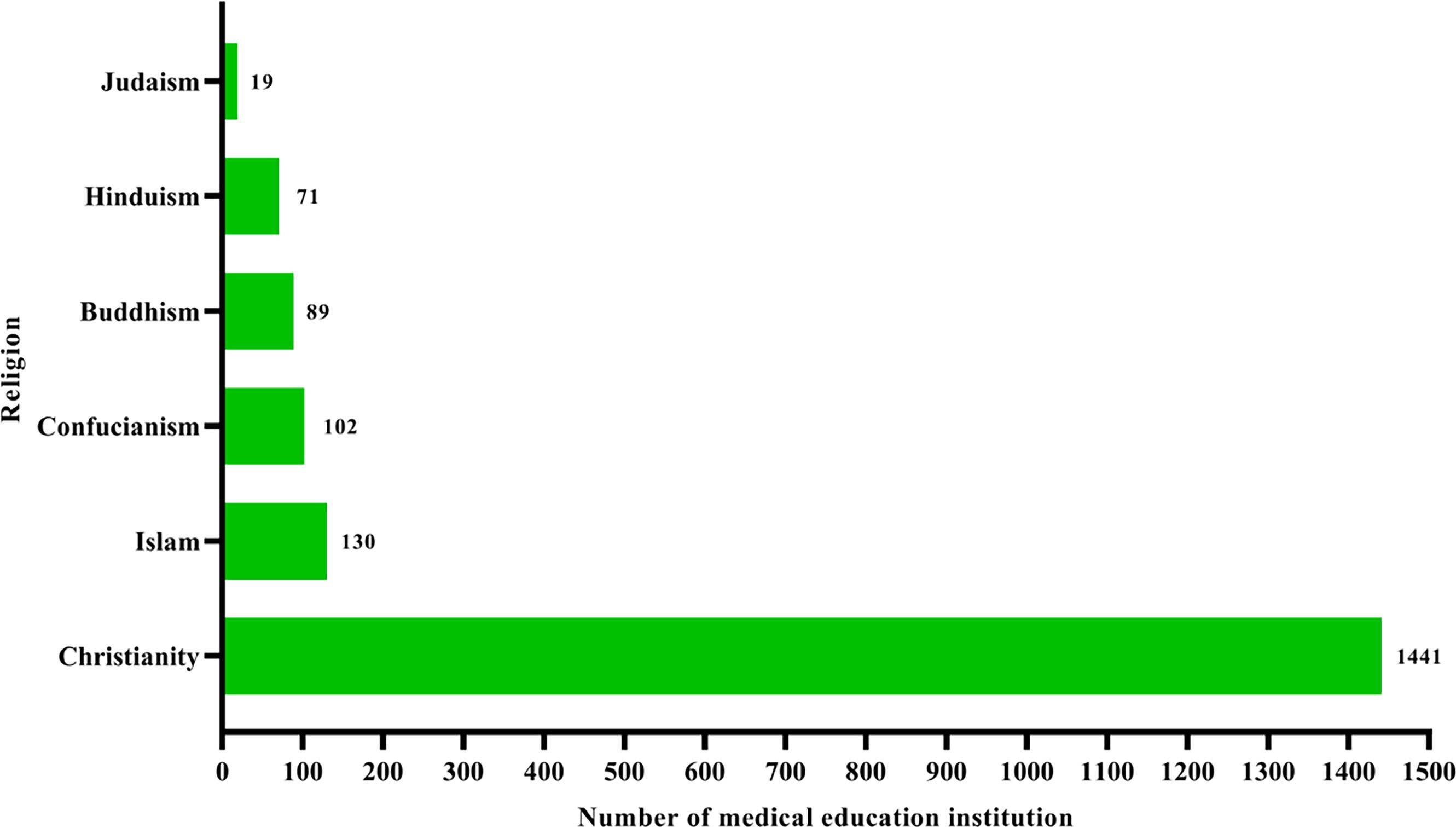

Supplement: Multimedia Appendix 2 [file mededu-v11-e71935-s002.jpeg]
